# Constructing a nomogram for forecasting incomplete clinical recovery following unilateral biportal endoscopy in lumbar disc herniation cases

**DOI:** 10.3389/fsurg.2026.1835904

**Published:** 2026-05-29

**Authors:** Haonan Lu, Tao Tang, Shenliang Chen, Zhifeng Cheng, Jiafeng Hong, Hao Xu, Qingmei Tang, Bo Hu

**Affiliations:** 1 Department of Spinal Surgery, Jiangxi Province Hospital of Integrated Chinese and Western Medicine, Nanchang, China; 2 Graduate School, Jiangxi University of Chinese Medicine, Nanchang, China

**Keywords:** incomplete clinical improvement, lumbar disc herniation (LDH), nomogram model, risk factors, unilateral biportal endoscopy (UBE)

## Abstract

**Background:**

Unilateral dual channel endoscopy (UBE) has unique advantages in lumbar degenerative lesions, but some patients show incomplete clinical symptom relief postoperatively, prolonging hospital stay and delaying recovery. In the existing studies, there is no design study on the nomogram model of incomplete clinical improvement after UBE surgery for lumbar disc herniation. Therefore, in order to accurately predict the occurrence of incomplete clinical improvement after UBE, this study constructed a nomogram model, which has significant predictive ability and clinical utility, and can provide great help for clinical decision-making.

**Methods:**

This study was based on the data of UBE operation in Jiangxi Province Hospital of Integrated Chinese & Western Medicine for lumbar disc herniation from January 2021 to December 2024. In this study, 290 patients’ data were divided into training set and validation set according to the ratio of 7:3. In the training set, we screened out the risk factors related to clinical research through statistical analysis, further identified the relevant independent risk factors through multiple logistic regression, and constructed a nomogram model based on the results. In the verification process of the model, ROC curve, decision curve DCA and calibration curve are used to determine whether it has reliability in the actual scene.

**Results:**

Through statistical analysis, this study screened out four indicators that were not completely related to the clinical improvement after UBE and built a nomogram model based on this: Increased body mass index (BMI), higher preoperative visual analogue scale (VAS-B) for back pain, increased preoperative Oswestry disability index (ODI) score, and presence of facet joint osteoarthritis (FJOA). The C-index of the training set and the validation set were 0.86 [95% confidence interval (CI): 0.76–0.95] and 0.92 (95% CI: 0.85–0.99).

**Conclusions:**

The key predictors identified in this study included high body mass index (BMI), elevated preoperative visual analogue scale (VAS-B) for back pain, elevated preoperative Oswestry disability index (ODI), and the presence of small joint osteoarthritis (FJOA). The prediction ability of this model is excellent, which can help clinicians make clinical decisions to a certain extent, so that timely intervention can be carried out to optimize the postoperative outcome.

## 1 Introduction

### 1.1 Research background

The main symptom of lumbar disc herniation (LDH) is irritation or compression of the nerve roots or cauda equina. This irritation and compression originates from the herniated disc tissue and develops on the pathological basis of lumbar disc herniation. The typical manifestations of patients include low back pain, numbness of lower limbs, muscle strength decline of lower limbs, radiation pain of lower limbs, and abnormal urination and defecation function. These symptoms significantly impair the functional quality of life of patients (1). As a minimally invasive surgical method for treating spinal diseases, unilateral dual channel endoscopy (UBE) has many technical advantages: small surgical trauma, clear surgical field, less intraoperative blood loss, and significant performance in reducing the risk of nerve injury and postoperative infection rate. Similarly, it can effectively shorten the postoperative recovery period of patients, so it is a high-quality choice for the treatment of spinal diseases (2).

### 1.2 Research status and limitations

Although spinal endoscopy has become mature in the treatment of lumbar disc herniation (LDH), the accurate evaluation of postoperative prognosis is still a major challenge in clinical practice. Most of the existing studies focus on the prognostic factors of percutaneous endoscopic lumbar discectomy (PELD) and transforaminal endoscopic discectomy (PETD). It has been confirmed that high body mass index (BMI), high preoperative low back pain visual analogue scale (vas-b), Modic changes, Pfirrmann classification and previous discectomy history are the key risk factors for incomplete clinical improvement after PELD (3, 4). These studies provide an important reference for evaluating the prognosis after spinal endoscopic surgery. However, whether its conclusion is applicable to unilateral dual channel endoscopy (UBE) technology still needs to be verified.

### 1.3 Research objectives and significance

This study aimed to recognize independent risk factors and to compile a nomogram. It will do this by analyzing the patient's clinical data. These patients underwent unilateral double portal vein endoscopic (UBE) surgery. This model helps clinicians identify high-risk patients, optimize preoperative decisions (e.g., modify surgical protocols, enhance perioperative care), and improve patient postoperative outcomes.

## 2 Materials and methods

### 2.1 Study participants

#### 2.1.1 Inclusion criteria

① Definitive diagnosis of lumbar disc herniation (LDH) confirmed; ② Received unilateral biportal endoscopic (UBE) lumbar discectomy following the ineffectiveness of conservative treatment; ③ Complete clinical and follow-up data; ④ Stable vital signs.

#### 2.1.2 Exclusion criteria

① Recurrent lumbar disc herniation; ② Spinal tumors; ③ Past medical history of lumbar spine surgery (for example, spinal fusion surgery); ④ History of mental illness; ⑤ Patients with preoperative spinal instability, lumbar spondylolisthesis and the need for lumbar fusion surgery were excluded; ⑥The follow-up data were missing or incomplete. Ethical statement: This study was approved by the ethics committee of Jiangxi provincial integrated traditional Chinese and Western Medicine Hospital Affiliated to Jiangxi University of traditional Chinese medicine, and all patients signed the informed consent form.

### 2.2 Definition of outcome

The outcome variables were defined as two categories: incomplete clinical improvement group (Group I) and complete improvement group (Group C). The definition of “incomplete clinical improvement” includes any of the following items: ① The degree of postoperative lumbocrural pain measured by VAS visual analogue scale did not reach the minimum clinically significant difference (MCID) threshold improvement; ② Oswestry disability index (ODI) was used to assess the degree of pain related dysfunction, which also did not reach MCID (5); ③ Evaluation via the modified MacNab criteria was classified as “fair” or “poor”.

### 2.3 Data collection

The clinical characteristics of patients who met the research criteria were collected, including: age (years), gender (male/female), body mass index (BMI), complications (such as diabetes, hypertension), smoking/drinking history, preoperative Oswestry Disability Index (ODI) scores, preoperative Visual Analog Scale for Back Pain (VAS-B) scores, preoperative Visual Analog Scale for Leg Pain (VAS-L) scores, surgical approach (interlaminar/transforaminal), surgical segment, and follow-up duration. Preoperative lumbar spine assessment parameters via x-ray, CT, and MRI included: disc height index (DHI) (6), lumbar lordosis (LL), sacral slope (SS), sagittal range of motion (SROM), facet joint orientation (FO), facet joint tropism (FT), type of disc herniation, facet joint osteoarthritis (FJOA), Modic changes of vertebral endplate inflammation (7), Pfirrmann classification of disc degeneration (8), and vacuum disc phenomenon.

All imaging assessments and clinical outcome evaluations were independently completed by two senior spine surgeons and two physician assistants. The assessors were blinded to the baseline data, grouping and postoperative outcomes of patients by single blind method. All imaging data and medical records have been de-identified before evaluation. If there is disagreement in the evaluation opinion, another senior spinal surgeon who also blinded all clinical data will make the final decision. Cohen kappa coefficient (*κ*) was used to evaluate the intergroup reliability of the key imaging grading system. Pfirrmann classification *κ*=0.842, Modic changes *κ*=0.827, and FJOA grading *κ*=0.856, all indicating excellent consistency (*κ* > 0.80). All results suggested excellent consistency between groups (*κ* > 0.80). The evaluation indexes included visual analogue scale for lower limb pain (vas-l), visual analogue scale for low back pain (vas-b), Oswestry (ODI) and modified macnab efficacy criteria.

### 2.4 Surgical procedure

All operations were performed under standardized conditions. When using unilateral double channel endoscopy (UBE), the surgical approach is determined according to the specific pathological characteristics and anatomical structure of the patients. There are two options, including interlaminar approach or paraspinal approach. With the aid of fluoroscopy of the C-arm machine, definite the positioning of the target surgical segment on the body surface, and mark the entry points of the two channels. Insert the puncture needle respectively, and adjust its position through repeated fluoroscopy. Then the guide wire was introduced and a 0.5–1 cm skin incision was made at two marked points along the guide wire. Two independent surgical channels were established by using the sequence expander: the observation channel and the working channel were established in turn. After the corresponding working cannula was inserted, the 30° endoscope was inserted through the endoscopic port. Saline continuous lavage was used to control bleeding and maintain a clear field of vision. Surgical instruments such as grinding drill and bone biting forceps were used for spinal canal decompression. The position of the working cannula in the working channel can be flexibly adjusted according to the surgical field of vision. The herniated nucleus pulposus tissue was accurately removed with intervertebral disc forceps, and the thickened ligamentum flavum was removed. After confirming that the nerve root and dural sac have been fully decompressed and there is no active bleeding in the surgical area, remove the two channels of the endoscope and the working cannula in turn. Finally, two skin incisions were sutured with suture.

### 2.5 Statistical analysis

Shapiro Wilk test was used to evaluate the normality of the data. Chi square (x ^2^) test, Fisher exact test and nonparametric rank sum test were used to compare the data between groups. A total of 290 patients who met the criteria were included in this study and divided according to the 7:3 ratio commonly used in clinical prediction model research: the sample size of the training set=290 × 70% = 203 cases, and the sample size of the validation set=290 × 30% = 87 cases; The classification basis conforms to the international general practice of clinical prediction model (nomogram). The training set ≥ 70% to ensure the modeling efficiency, and the validation set ≥ 30% to ensure the validation reliability; Meet the sample size requirements of logistic regression (number of events ≥ 40, number of predictors=4, sample size/number of factors > 10); The balance test of the baseline data of the two groups showed that *p* > 0.05, there was no statistical difference, and the grouping was reliable. There was no significant difference in the incidence of incomplete improvement between the two groups. According to this, binary logistic regression analysis was carried out to determine independent risk factors, and the nomogram model was constructed. In the validation phase, SPSS software was used to statistically analyze the two groups of data, calculate the area under the receiver operating characteristic (ROC) curve (AUC) (4), and then evaluate the discrimination ability of the model, that is, the ability to distinguish between patients with complete clinical improvement and patients with incomplete clinical improvement. The calibration curve and Hosmer lemeshow goodness of fit test were used to evaluate the calibration degree, and the clinical utility of the model was evaluated with the help of decision curve analysis (DCA) (4). *P*-value < 0.05 was considered statistically significant. All patients completed the follow-up, with a median follow-up time of 12.0 months (range, 6.0–24.0 months), and the follow-up rate was 100%. Before multivariate logistic regression analysis, variance inflation factor (VIF) was used to carry out multicollinearity diagnosis. The body mass index (BMI), preoperative VAS-B score, preoperative ODI score and FJOA variance inflation factor values were 1.32, 1.45, 1.61 and 1.27, respectively. All variance inflation factor values were less than 2.0, suggesting that there was no obvious multicollinearity between the respective variables.

## 3 Results

### 3.1 Baseline characteristics of patients

From January 2021 to December 2024, 290 patients with lumbar disc herniation (LDH) who met the research conditions were selected, and these patients underwent unilateral dual channel endoscopic (UBE) surgery. Our study found that 47 cases (16.2%) did not meet the standard of clinical improvement. In the training set (*n* = 203), 33 cases of postoperative clinical improvement did not meet the expectations, and were divided into group I (incomplete improvement) according to the definition of this study; The remaining 170 patients with postoperative pain and dysfunction were significantly relieved, so they were divided into group C (completely improved). In the validation set (*n* = 87), 14 of the 87 patients with incomplete postoperative clinical improvement were classified into group I, and 73 patients were significantly relieved of postoperative pain and dysfunction, and were classified into group C. Data conforming to normal distribution are expressed as mean ± standard deviation, and data not normally distributed are expressed as percentiles (Table 1).

**Table 1:** Baseline characteristics of the training and validation sets.

Variables	Training set				Validation set			
	Total(*n* = 203)	Complete(*n* = 170)	Incomplete (*n* = 33)	*p*-value	Total(*n* = 87)	Complete(*n* = 73)	Incomplete (*n* = 14)	*p*-value
Age [mean (SD)]	56.48 ± 9.57	56.05 ± 9.52	58.70 ± 9.68	0.147	57.15 ± 9.47	56.85 ± 10.00	58.71 ± 6.01	0.356
Sex (Female, %)	115 (57)	95 (55.9)	20 (60.6)	0.616	42 (48.3)	34 (46.6)	8 (57.1)	0.469
Duration of symptom (months)	6.00 (3.00，10.00)	6.00（3.00，10.00）	8.00 (1.50,10.00)	0.658	6.00 (2.00，10.00)	6.00（2.00，10.00）	8.00 (5.50,12.00)	0.105
Current smoking (*n*, %)	58 (28.6)	49 (28.8)	9 (27.3)	0.857	17 (19.5)	13 (17.8)	4 (28.6)	0.574
Current drinking (*n*, %)	52 (25.6)	42 (24.7)	10 (30.3)	0.5	20 (23.0)	15 (20.5)	5 (35.7)	0.374
Diabetes (*n*, %)	31 (15.3)	27 (15.9)	4 (12.1)	0.583	25 (28.7)	22 (30.1)	3 (21.4)	0.786
Hypertension (*n*, %)	58 (28.6)	49 (28.8)	9 (27.3)	0.857	32 (36.8)	27 (37.0)	5 (35.7)	0.928
Surgical segment (n, %)				0.953				0.721
L3-4	19 (9.4)	16 (9.4)	3 (9.1)		8 (9.2)	6 (8.2)	2 (14.3)	
L4-5	140 (70.0)	118 (69.4)	22 (66.7)		60 (69.0)	51 (69.9)	9 (64.3)	
L5-S1	44 (21.7)	36 (21.2)	8 (24.2)		19 (21.8)	16 (21.9)	3 (21.4)	
BMI [mean (SD)]	24.81 ± 2.05	24.51 ± 1.69	26.36 ± 2.92	0.001	24.55 ± 1.38	24.29 ± 1.08	25.91 ± 1.94	0.009
Preoperative clinical score								
VAS-B	7.00 (6.00，8.00)	7.00 (6.00,8.00)	8.00 (7.00,8.00)	<0.001	7.00 (6.00，8.00)	7.00 (6.00,8.00)	7.00 (6.00,8.00)	0.834
VAS-L	6.00 (5.00，7.00)	6.00 (5.00,7.00)	7.00 (6.00,8.00)	0.003	6.00 (5.00，7.00)	6.00 (5.00,8.00)	6.00 (5.00,6.25)	0.746
ODI	59.00 (52.00，64.00)	57.00 (50.00,63.00)	64.00 (60.50,70.50)	<0.001	56.00 (50.00，66.00)	54.00 (49.50,63.50)	63.00 (60.75,66.00)	0.003
Surgicalapproach (*n*, %)	81（39.9）	66 (38.8)	15 (45.5)	0.477	35（40.2）	30 (41.1)	5 (35.7)	0.707
Type of disc herniation (*n*, %)				0.819				0.785
Protrusion	148（72.9）	125 (73.5)	23 (69.7)		60（69.0）	50 (68.5)	10 (71.4)	
Extrusion	39（19.2）	30 (17.6)	9 (27.3)		25（28.7）	21 (28.8)	4 (28.6)	
Sequestration	16（7.9）	15 (8.8)	1 (3.0)		2（2.3）	2 (2.7)	0 (0)	
MODIC (*n*, %)				0.028				0.593
0	133（65.5）	117 (68.8)	16 (48.5)		58（66.7）	50 (68.5)	8 (57.1)	
1	19（9.4）	14 (8.2)	5 (15.2)		9（10.3）	6 (8.2)	3 (21.4)	
2	49（24.1）	38 (22.4)	11 (33.3)		18（20.7）	15 (20.5)	3 (21.4)	
3	2（1.0）	1 (0.6)	1 (3.0)		2（2.3）	2 (2.7)	0 (0)	
DHI	0.31 (0.27，0.34)	0.31 (0.28,0.33)	0.31 (0.25，0.36)	0.168	0.32 (0.28，0.35)	0.32 (0.28,0.36)	0.32 (0.25，0.34)	0.254
SROM	3.30 (2.00，6.30)	3.35 (2.00,6.30)	3.00 (2.04,5.10)	0.514	3.00 (1.98，6.00)	3.10 (1.97,6.35)	2.70 (1.82,4.80)	0.267
LL [mean (SD)]	32.94 ± 9.62	32.56 ± 9.62	34.88 ± 9.55	0.206	31.59 ± 9.45	30.40 ± 8.79	37.83 ± 10.64	0.006
SS	27.90 (24.00，32.80)	28.00 (23.50,33.62)	27.50 (25.10,29.75)	0.211	27.20 (23.50，31.00)	26.20 (23.50,30.25)	29.10 (24.10,33.50)	0.162
VDP (*n*, %)	39（19.2）	36 (21.2)	3 (9.1)	0.107	24（27.6）	22 (30.1)	2 (14.3)	0.374
FO	3.72 (2.30，6.60)	3.79 (2.30,6.60)	3.53 (2.40,6.75)	0.678	3.70 (2.20，6.60)	3.59 (1.77,6.60)	5.65 (3.31,6.63)	0.208
FT	3.35 (2.69，4.07)	3.34 (2.61,4.03)	3.42 (2.84,4.27)	0.256	3.29 (2.67，3.99)	3.29 (2.56,4.01)	3.39 (2.87,3.99)	0.474
Pfirmann (*n*, %)				0.233				0.911
1	35（17.2）	30 (17.6)	5 (15.2)		13（14.9）	13 (17.8)	0 (0)	
2	39（19.2）	29 (17.1)	10 (30.3)		19（21.8）	15 (20.5)	4 (28.6)	
3	47（23.2）	38 (22.4)	9 (27.3)		20（23.0）	14 (19.2)	6 (42.9)	
4	41（20.2）	37 (21.8)	4 (12.1)		16（18.4）	13 (17.8)	3 (21.4)	
5	41（20.2）	36 (21.2)	5 (15.2)		19（21.8）	18 (24.7)	1 (7.1)	
FJOA (*n*, %)	94（46.3）	71 (41.8)	23 (69.7)	0.003	38（43.7）	31 (42.5)	7 (50.0)	0.603

The median follow-up time was 12.0 months (range 6.0–24.0 months).

### 3.2 Statistical analysis

Five factors were significantly correlated with incomplete clinical improvement (*p* < 0.05). Through binary logistic regression analysis, we found higher body mass index (BMI), preoperative high back pain visual analogue scale (VAS-B), preoperative high visual analogue scale for leg pain (VAS-L), preoperative high Oswestry disability index (ODI) and small joint osteoarthritis (FJOA) were risk factors for incomplete clinical improvement after UBE surgery. These results are presented in Table 2.

**Table 2:** .Binary logistic regression analysis of risk factors for incomplete clinical improvement after UBE

Variable	OR	95% CI	*P*-value
Age	1.028	0.993–1.063	0.114
Sex (Female 5s. Male)	1.302	0.407–1.449	0.415
Duration of symptom	1.013	0.978–1.050	0.473
Current smoking (Yes vs. No)	0.896	0.444–1.806	0.759
Current drinking (Yes vs. No)	0.654	0.331–1.292	0.221
Diabetes (Yes vs. No)	1.443	0.610–3.418	0.404
Hypertension (Yes vs. No)	1.073	0.543–2.210	0.840
Surgical segment (reference: L3–4)	—	—	—
L4–5 vs. L3–4	0.807	0.284–2.292	0.687
L5–S1 vs. L3–4	0.931	0.289–2.995	0.904
BMI	1.663	1.381–2.002	**<0**.**001**
Preoperative VAS–B	1.647	1.222–2.221	**0**.**001**
Preoperative VAS–L	1.258	1.030–1.536	**0**.**024**
Preoperative ODI	1.092	1.055–1.131	**<0**.**001**
Surgical approach	0.882	0.468–1.660	0.696
Type of disc herniation (reference: Protrusion)	—	—	—
Extrusion vs. Protrusion	1.352	0.662–2.759	0.408
Sequestration vs. Protrusion	0.312	0.040–2.425	0.266
Modic degeneration (Yes vs. No)	0.626	0.326–1.201	0.159
DHI	0.111	0.000–50.532	0.481
SROM	0.909	0.801–1.032	0.142
LL	0.971	0.908–1.037	0.377
SS	0.982	0.945–1.021	0.367
VDP (Yes vs. No)	2.634	0.995–6.968	0.051
FO	1.064	0.952–1.190	0.271
FT	1.349	0.914–1.990	0.131
Pfirrmann grade (reference: Grade 1)	—	—	—
Pfirrmann degeneration (Moderate-severe vs. Mild)	0.822	0.437–1.557	0.547
FJOA (Yes vs. No)	3.561	1.278–9.923	**0**.**015**

Bold values indicate statistical significance (*P* < 0.05).

### 3.3 Construction and validation of the nomogram model

**Figure 1:**
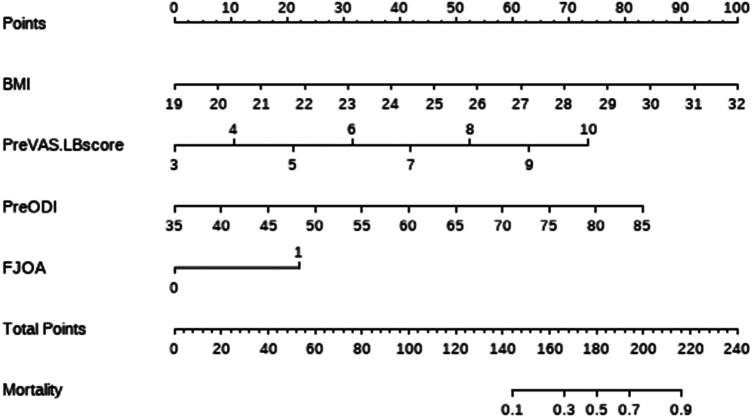
Nomogram model for predicting incomplete clinical improvement after unilateral biportal endoscopy in lumbar disc herniation cases.

In the final nomogram model, variable types were defined as follows as shown in Figure 1: The following variables were treated as continuous variables and entered into the model without dichotomization: body mass index (BMI), preoperative VAS-B score, and preoperative ODI score. Facet joint osteoarthritis (FJOA) was included as a dichotomous variable (0 = absent, 1 = present).

The final nomogram was constructed based on four independent risk factors identified by multivariate binary logistic regression analysis. The regression equation of the prediction model is as follows:

Logit(P)=−6.728 + 0.456×BMI + 0.644×PreVAS-B + 0.101×PreODI + 1.270×FJOA,

Where P indicates the possibility of clinical complete recovery after UBE.

The odds ratio (or) and 95% confidence interval (CI) of each predictor in the nomogram are as follows:

- BMI: OR = 1.578, 95%CI = 1.246–1.998, P＜0.001;
- Preoperative VAS-back pain (PreVAS-B): OR = 1.904, 95%CI = 1.114–3.253, *P* = 0.018;
- Preoperative ODI score: OR = 1.106, 95%CI = 1.045–1.171, *P* = 0.001;
- Facet joint osteoarthritis (FJOA): OR = 3.561, 95%CI = 1.278–9.923, *P* = 0.015.

In the nomogram, each predictor is assigned a corresponding weighted score according to its regression coefficient. The total score of each patient is obtained by summing the scores of each variable, according to which the predicted probability of incomplete clinical improvement can be calculated. The c-index of the model is 0.82 (95% confidence interval: 0.76–0.88), and the calibration curve shows a good agreement between the predicted probability and the actual observation.

**Figure 2:**
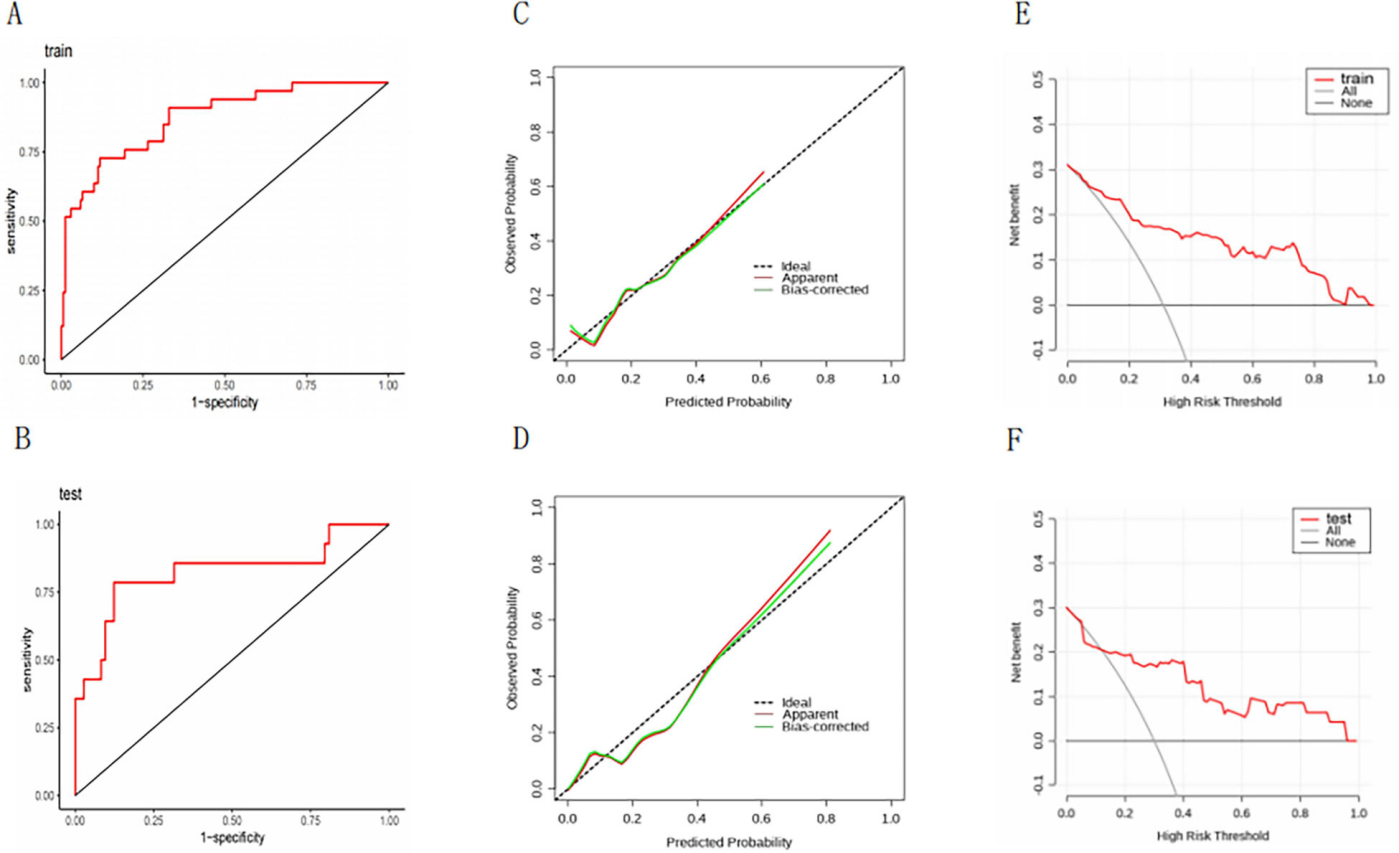
Calibration curve DCA decision curve ROC curve (AUC) of training set and verification set. **(A,B)** The area under the receiver operating characteristic curve (ROC) (AUC) of training set and verification set. **(C,D)** The calibration curve analysis of training set and verification set. **(E,F)** The decision curve analysis (DCA) of training set and verification set.

The discrimination performance of the nomogram was assessed by receiver operating characteristic (ROC) curve analysis. The AUC values were clearly reported in the text:

- Training set: AUC = 0.86 (95%CI: 0.81–0.92), corresponding to Figure 2A;
- Validation set: AUC = 0.82 (95%CI: 0.67–0.97), corresponding to Figure 2B.

The calibration curve analysis showed that the model prediction results were highly consistent with the real clinical observation data (Figures 2C,D). In addition, the Hosmer lemeshow goodness of fit test yielded a *p*-value of 0.417, confirming that the nomogram model has excellent calibration performance. The clinical utility of the model was verified by decision curve analysis (DCA): the training set presented a positive net benefit in the range of 1%–97% probability threshold (Figure 2F), while the validation set achieved a positive net benefit in the range of 2%–95%. In the validation set, the nomogram had significant discrimination efficiency, with an AUC value of 0.86 (95%ci:0.79–0.93). In conclusion, the nomogram model developed in this study shows excellent prediction ability.

### 3.4 Blinding procedure for imaging assessment and outcome analysis

In order to minimize potential selection bias and observation bias, the blind operation process of image evaluation and outcome analysis strictly adopts a double-blind and independent evaluation scheme in the process of image evaluation and clinical outcome analysis: Blind treatment of imaging evaluation: all preoperative imaging evaluation indicators [including Modic score change, Pfirrmann grade, facet osteoarthritis (FJOA) score, intervertebral disc height index, sagittal parameters, etc.] were independently measured by two senior orthopedic doctors who were unaware of the patient's baseline information, surgical records, and postoperative results. All images were identified in the past and only the random serial number was retained. Any disagreement was adjudicated by a third senior spine surgeon who also remained blind to all clinical information until consensus was reached.

Blinding of outcome assessment: postoperative clinical outcomes [including visual analogue scale (VAS) score, objective disease index (ODI) score, and modified macnab criteria] were assessed by two independent research assistants who were blinded to preoperative imaging findings, baseline characteristics, and complete/incomplete improvement grouping.

## 4 Discussion

Unilateral dual channel spinal endoscopic (UBE) decompression technology has significant differential advantages in the field of lumbar disease intervention (9). However, in actual clinical practice, it is common to see that the clinical improvement after UBE is not complete, and the mechanism behind it is still not completely clear. Through this retrospective study, we systematically evaluated the clinical recovery of patients undergoing UBE surgery, and then recognized the core risk factors closely related to the poor prognosis after surgery. The research data showed that the overall proportion of achieving complete improvement after surgery was 83.7%, which strongly supported the clinical effectiveness of UBE technology for treating lumbar disc herniation (LDH) (10). After in-depth analysis, we found that higher body mass index (BMI), higher preoperative lumbar visual analogue scale (VAS-B) and Oswestry disability index (ODI), and the presence of spondyloarthroid osteoarthritis (FJOA) were independent risk factors for inadequate clinical improvement after UBE. Drawing on the above findings, we constructed a nomogram prediction model. The model shows excellent prediction efficiency after testing, and is expected to become a practical tool for preoperative risk classification, and provide an important reference for clinical diagnosis and treatment decisions. Obesity has been recognized as a risk factor affecting the clinical efficacy of lumbar surgery in many reports (11–14). Compared with non obese patients, obese patients are less likely to improve after the intervention of intervertebral disc herniation surgery (14). In this study, BMI was positively correlated with the outcome of incomplete improvement after surgery. Obese patients are more likely to have general or poor clinical efficacy after lumbar surgery (15). High BMI means shorter height, heavier weight, and higher intervertebral disc load; at the same time, obese patients are more likely to have changes in posture and flexibility of the thoracolumbar spine, changing the biomechanics of the spine and increasing its mechanical load (16). That is to say, weight management has a certain effect on inhibiting the process of inflammatory response, so as to relieve pain. A study investigating the concentration of glycosaminoglycans (GAGs) in the nucleus pulposus and intervertebral disc (IVD) degeneration indicated that a decrease in GAG concentration triggers IVD degeneration (17). Thus accelerating inflammation and extracellular matrix breakdown, releasing neurotrophins and ultimately leading to peripheral neuronal innervation with abnormal excitability, the increased sensitization of these neurons plays a prominent role in pain chronicity (18). Compared with simple low back pain, individuals with low back pain related leg pain had worse quality of life (19). The improvement of these patients after surgery is indeed not satisfactory. Combined with our study, higher preoperative VAS-B and ODI may reflect high levels of inflammatory factors, thus causing immune response (18). If the patient has spondyloarthroid osteoarthritis, the possibility that the pain has not been completely improved after surgery will be greatly increased. FJOA is the result of spinal degeneration and is inseparable from low back pain (20). The three joint complex composed of paired facet joints and intervertebral discs is more likely to develop FJOA due to changes in structural integrity, such as degeneration of intervertebral discs or abnormal joint alignment (21). Lgarashi A and others pointed out that the inflammatory factors produced in FJ tissues may diffuse into the vertebral cavity through the lateral aspect of the ventral facet joint capsule, and the inflammatory factors repeatedly stimulate the surrounding tissues to cause pain. Therefore, in the whole treatment cycle, non steroidal anti-inflammatory drugs are a good treatment option (22).

### 4.1 Study limitations

The limitations of this study are reflected in the following aspects: (1) This study is a retrospective study, which may have potential selection bias and information bias. In order to reduce selection bias as much as possible, all consecutive cases who met the inclusion criteria and underwent UBE surgery from January 2021 to December 2024 were included in this study. No artificial random exclusion was performed. Clear and unified inclusion and exclusion criteria were used throughout the study. Prospective cohort study design will be adopted in the future to further avoid selection bias and improve the level of research evidence. (2) First, this study was conducted in a single-center setting. Although internal validation was performed, no external validation was conducted. (3) The short follow-up time may lead to insufficient data integrity and reduced data representativeness. (4) Intraoperative variables such as operation time, intraoperative blood loss, decompression range were not included in the model construction. All patients underwent standardized unilateral dual channel endoscopic (UBE) surgery by the same surgical team, making the intraoperative related parameters highly consistent. More importantly, intraoperative related indicators cannot be obtained preoperatively, which is contrary to the original intention of preoperative prediction research. This nomogram model only uses preoperative related variables for preoperative risk prediction, so as to ensure its clinical applicability and practical application value. (5) In addition, this model did not include indicators of postoperative activity status and work-related needs, because such indicators could not be obtained preoperatively and could not be used for preoperative risk stratification assessment. The above factors may have an impact on postoperative outcomes, which is worthy of further exploration in the follow-up prospective study.

### 4.2 Cost-effectiveness and practical implementation barriers for clinical use

The clinical application of this nomogram model needs to comprehensively consider the cost-effectiveness and practical implementation obstacles. All predictors (BMI, preoperative VAS-B score, preoperative ODI score, and FJOA) included in the model were derived from preoperative routine clinical evaluation and magnetic resonance examination, without additional cost, additional examination items, or professional equipment. Therefore, the nomogram has excellent cost-effectiveness, is suitable for clinical popularization, and will not increase the medical economic burden of patients.

Nevertheless, there are still several practical obstacles in the actual clinical application of this model. First, the popularization and application of the model rely on the standardization of imaging grading and outcome evaluation, while there are differences in the relevant evaluation standards between different medical institutions. Second, this nomogram is based on a single center retrospective cohort study. There are differences in patient composition, surgical diagnosis and treatment experience, and perioperative management programs in different regions, which may affect the application efficiency of the model. Third, the acceptance and application proficiency of the model by clinical medical staff will also affect the implementation efficiency of the prediction model. The above existing obstacles need to be further improved and solved through multi center prospective verification and standardized training.

### 4.3 External validation feasibility and validation schedule

External validation is essential to ensure the generalization and clinical applicability of the nomogram model.

#### 4.3.1 External validation feasibility

It is feasible to carry out multi center external verification of this nomogram. First of all, the predictors (body mass index, preoperative back visual analog pain score, preoperative Oswestry dysfunction index score, and small joint osteoarthritis grade) included in the model are conventional clinical and imaging indicators, which can be conveniently and uniformly collected in different medical institutions without additional equipment and complex processes. Secondly, the surgical method of unilateral dual channel endoscopic technology in the treatment of lumbar disc herniation has been popularized and standardized in many spine centers, which can ensure the consistency of perioperative management and outcome evaluation. Third, the definition of study outcome (poor clinical improvement) is based on the universal evaluation scale (visual analogue scale, Oswestry disability index, modified macnab standard), which can ensure that different institutions adopt a unified evaluation standard. In conclusion, the nomogram is qualified for external validation in other hospitals.

#### 4.3.2 External validation schedule

Months 1–3: establish a multi center scientific research cooperation system and complete the formulation of data collection plan.

Months 4–9: collect clinical research data from at least two external medical institutions.

Months 10–12: carry out external validation, including model discrimination, calibration and clinical utility evaluation, and complete the writing of follow-up study report.

This link can further verify the stability and portability of the model, and promote the popularization and application of the model in clinical diagnosis and treatment.

## 5 Conclusion

Our nomogram developed herein showed excellent performance in predicting the risk factors of incomplete improvement after UBE, among which BMI, preoperative VAS-B, preoperative ODI and FJOA were identified as the key predictive factors after analysis. It can provide valuable reference for clinical decision-making, optimize the allocation of medical resources, and guide clinical decision.

## Data Availability

The original contributions presented in the study are included in the article/Supplementary Material, further inquiries can be directed to the corresponding author.

## References

[1] Wei FL Zhou CP Zhu KL Du MR Liu Y Heng W. Comparison of different operative approaches for lumbar disc herniation: a network meta-analysis and systematic review. Pain Physician. (2021) 24(4):E381–92. 34213864

[2] Kim KR Park JY. The technical feasibility of unilateral biportal endoscopic decompression for the unpredicted complication following minimally invasive transforaminal lumbar interbody fusion: case report. Neurospine. (2020) 17(Suppl 1):S154–9. 10.14245/ns.2040174.087 32746529 PMC7410383

[3] Jia M Sheng Y Chen G Zhang W Lin J Lu S. Development and validation of a nomogram predicting the risk of recurrent lumbar disk herniation within 6 months after percutaneous endoscopic lumbar discectomy. J Orthop Surg Res. (2021) 16(1):274. 10.1186/s13018-021-02425-2 33882995 PMC8059294

[4] Li T Jiang Q Yang G Ding Z Ding Y. Development of a nomogram model for predicting incomplete clinical improvement after percutaneous endoscopic lumbar discectomy. J Clin Neurosci. (2025) 140:111508. 10.1016/j.jocn.2025.111508 40684580

[5] Jitpakdee K Liu Y Kim YJ Kotheeranurak V Kim JS. Factors associated with incomplete clinical improvement in patients undergoing transforaminal endoscopic lumbar discectomy for lumbar disc herniation. Eur Spine J. (2023) 32(8):2700–8. 10.1007/s00586-023-07636-1 36917301

[6] Kim KT Park SW Kim YB. Disc height and segmental motion as risk factors for recurrent lumbar disc herniation. Spine (Phila Pa 1976). (2009) 34(24):2674–8. 10.1097/BRS.0b013e3181b4aaac 19910771

[7] Jensen TS Karppinen J Sorensen JS Niinimäki J Leboeuf-Yde C. Vertebral endplate signal changes (modic change): a systematic literature review of prevalence and association with non-specific low back pain. Eur Spine J. (2008) 17(11):1407–22. 10.1007/s00586-008-0770-2 18787845 PMC2583186

[8] Pfirrmann CW Metzdorf A Zanetti M Hodler J Boos N. Magnetic resonance classification of lumbar intervertebral disc degeneration. Spine (Phila Pa 1976). (2001) 26(17):1873–8. 10.1097/00007632-200109010-00011 11568697

[9] Deng Y Yang M Xia C Chen Y Xie Z. Unilateral biportal endoscopic decompression for symptomatic thoracic ossification of the ligamentum flavum: a case control study. Int Orthop. (2022) 46(9):2071–80. 10.1007/s00264-022-05484-0 35725953

[10] Yuan S Chen R Mei Y Fan N Wang T Wang A. Comparison of learning curves and clinical outcomes in unilateral biportal endoscopic spinal surgery versus percutaneous transforaminal endoscopic surgery: a cumulative sum analysis. J Pain Res. (2025) 18:631–42. 10.2147/jpr.S485283 39931426 PMC11809230

[11] Gepstein R Shabat S Arinzon ZH Berner Y Catz A Folman Y. Does obesity affect the results of lumbar decompressive spinal surgery in the elderly? Clin Orthop Relat Res. (2004) 426:138–44. 10.1097/01.blo.0000141901.23322.98 15346065

[12] Shahi P Subramanian T Araghi K Korsun MK Singh S Singh N. Class 2/3 obesity leads to worse outcomes following minimally invasive transforaminal lumbar interbody fusion. Spine J. (2025) 25(9):1985–96. 10.1016/j.spinee.2025.03.020 40154631

[13] Smith JS Shaffrey CI Glassman SD Carreon LY Schwab FJ Lafage V. Clinical and radiographic parameters that distinguish between the best and worst outcomes of scoliosis surgery for adults. Eur Spine J. (2013) 22(2):402–10. 10.1007/s00586-012-2547-x 23073746 PMC3555616

[14] Zhang Y Ding Z Wang J Guan L Liu Y Hai Y. Risk factors for recurrent lumbar disc herniation after unilateral biportal endoscopy: a retrospective study. Int Orthop. (2025) 49(8):1963–71. 10.1007/s00264-025-06577-2 40515760

[15] Quek CX Goh GS Tay AY Soh RCC. Minimally invasive versus open transforaminal lumbar interbody fusion in obese patients: a propensity score-matched study. Spine (Phila Pa 1976). (2024) 49(18):1294–300. 10.1097/brs.0000000000005042 38770556

[16] Ghezelbash F Shirazi-Adl A Plamondon A Arjmand N Parnianpour M. Obesity and obesity shape markedly influence spine biomechanics: a subject-specific risk assessment model. Ann Biomed Eng. (2017) 45(10):2373–82. 10.1007/s10439-017-1868-7 28608245

[17] Haneder S Apprich SR Schmitt B Michaely HJ Schoenberg SO Friedrich KM. Assessment of glycosaminoglycan content in intervertebral discs using chemical exchange saturation transfer at 3.0 tesla: preliminary results in patients with low-back pain. Eur Radiol. (2013) 23(3):861–8. 10.1007/s00330-012-2660-6 23052643

[18] Rudnik-Jansen I van Kruining Kodele S Creemers L Joosten B. Biomolecular therapies for chronic discogenic low back pain: a narrative review. JOR Spine. (2024) 7(3):e1345. 10.1002/jsp2.1345 39114580 PMC11303450

[19] Harrisson SA Stynes S Dunn KM Foster NE Konstantinou K. Neuropathic pain in low back-related leg pain patients: what is the evidence of prevalence, characteristics, and prognosis in primary care? A systematic review of the literature. J Pain. (2017) 18(11):1295–312. 10.1016/j.jpain.2017.04.012 28619698

[20] Suri P Hunter DJ Rainville J Guermazi A Katz JN. Presence and extent of severe facet joint osteoarthritis are associated with back pain in older adults. Osteoarthritis Cartil. (2013) 21(9):1199–206. 10.1016/j.joca.2013.05.013 PMC4018241 23973131

[21] Gellhorn AC Katz JN Suri P. Osteoarthritis of the spine: the facet joints. Nat Rev Rheumatol. (2013) 9(4):216–24. 10.1038/nrrheum.2012.199 23147891 PMC4012322

[22] Igarashi A Kikuchi S Konno S Olmarker K. Inflammatory cytokines released from the facet joint tissue in degenerative lumbar spinal disorders. Spine. (2004) 29(19):2091–5. 10.1097/01.brs.0000141265.55411.30 15454697

